# Bilateral Cavernous Carotid Aneurysms: Atypical Presentation of a Rare Cause of Mass Effect. A Case Report and a Review of the Literature

**DOI:** 10.3389/fneur.2018.00619

**Published:** 2018-08-02

**Authors:** Delia Gagliardi, Irene Faravelli, Luisa Villa, Guglielmo Pero, Claudia Cinnante, Roberta Brusa, Eleonora Mauri, Laura Tresoldi, Francesca Magri, Alessandra Govoni, Nereo Bresolin, Giacomo P. Comi, Stefania Corti

**Affiliations:** ^1^Dino Ferrari Centre, Neuroscience Section, Neurology Unit, Department of Pathophysiology and Transplantation, University of Milan, IRCCS Foundation Ca' Granda Ospedale Maggiore Policlinico, Milan, Italy; ^2^Neuromuscular Unit, Fondazione IRCCS Ca' Granda Ospedale Maggiore Policlinico, Dino Ferrari Center, University of Milan, Milan, Italy; ^3^Department of Neuroradiology, Ospedale Niguarda Ca' Granda, Milan, Italy; ^4^Neuroradiology Unit, Fondazione IRCCS Ca' Granda Ospedale Maggiore Policlinico, Milan, Italy; ^5^Ophthalmology Unit, Fondazione IRCCS Ca' Granda Ospedale Maggiore Policlinico, Milan, Italy

**Keywords:** bilateral cavernous carotid aneurysms, internal carotid artery, cranial nerves palsy, pseudomyasthenia, diplopia

## Abstract

Bilateral cavernous carotid aneurysms (CCAs) represent a rare medical condition that can mimic other disorders. We present a rare case of bilateral CCAs simulating an ocular myasthenia. A 76-year-old woman presented with a history of fluctuating bilateral diplopia and unilateral ptosis, which led to the suspicion of ocular myasthenia. Magnetic resonance imaging (MRI) and magnetic resonance angiography (MRA) of the brain showed the presence of large bilateral aneurysms of the carotid cavernous tract. After an unsuccessful attempt with steroid therapy, the patient underwent endovascular treatment, with mild improvement. Bilateral CCAs can cause pupil sparing third nerve palsies and other cranial neuropathies which can mimic signs and symptoms of disorders of the neuromuscular junction. Therefore, the possibility of a vascular lesion simulating ocular myasthenia should be considered especially in older patients.

## Introduction

Aneurysms of the cavernous tract of the carotid artery are a rare occurrence, with a reported prevalence varying from 0.3 to 1.4% of all intracranial aneurysms (1). These aneurysms are more common in women and can be idiopathic or due to trauma and infections. CCAs are also different from other intracranial aneurysms in terms of natural history and clinical presentation: they are often asymptomatic, have a low risk of life-threatening complications and are usually considered as benign lesions. Though uncommon, complications of CCAs have been reported, which include rupture into either the cavernous sinus, causing the formation of a carotido-cavernous fistula, or into the subarachnoid space, determining a subarachnoid hemorrhage. Other than spontaneous rupture, complications can derive from acute thrombosis or progressive compression of cranial nerves in the cavernous sinus.

Bilateral CCAs, owing to their unique location and proximity to important vital structures, can mimic other disorders. Here we present a rare case of bilateral CCAs simulating an ocular myasthenia and we reviewed the literature about previous cases of idiopathic unruptured bilateral CCAs, with particular reference to those mimicking other diseases.

## Case report

A 76-year-old woman presented to our clinic due to fluctuating diplopia, worse at the end of the day while watching television, which gradually developed for 8 months prior to presentation. She complained about binocular vision in primary gaze position, in vertical and right lateral gaze directions. Her medical history included breast cancer surgically resected without recurrence, smoldering multiple myeloma, bilateral cataract and osteoporosis.

Before presenting to us, she was evaluated as an outpatient.

Neurological examination showed right lateral rectus palsy causing an esotropia with gaze paretic nystagmus, moderate not fatigable ptosis and slight adduction deficit in the left eye. Pupils were round, equal and reactive to light. Vision was preserved on gross examination (the patient did not complain any vision loss) and confrontation visual field test resulted normal. Ophthalmological examination showed bilateral visual acuity deficit due to cataracts (20/63 in the right eye and 20/50 in the left eye), normal ocular pressure and normal fundus examination. The orthoptic measurements revealed an abduction deficit in right eye, a slight adduction deficit in left eye and a bilateral elevation deficit, greater in the left eye. The patient presented a 35 prism diopter esotropia in primary gaze. The strength of facial muscles, including orbicular oculi, was normal and Cogan's lid twitch sign was negative. The rest of physical examination was unremarkable.

Lab test included a complete cell blood count, liver, renal and thyroid function test, and returned normal.

Due to the clinical history of fluctuating symptoms, a screening for myasthenia gravis was undertaken: serum assays for acetylcholine receptor (AChR) antibodies and muscle-specific tyrosine kinase (MuSK) antibodies tested negative, electromyography (EMG) of the facial nerves with repetitive supramaximal stimulation and single-fiber EMG gave normal results.

**Figure 1:**
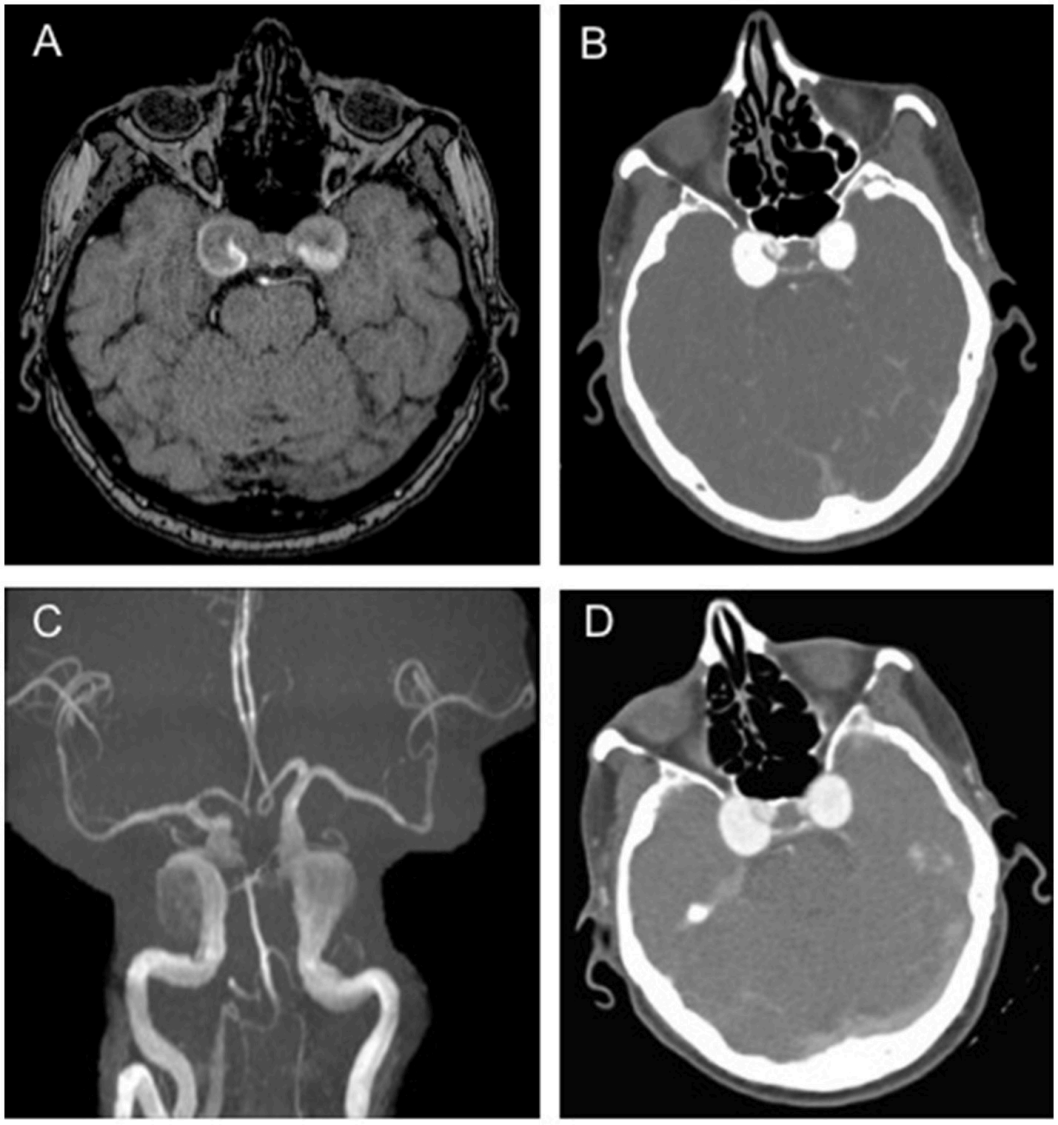
Brain imaging showing bilateral aneurysms.**(A)** T1-weighted axial brain magnetic resonance (MR) scan showing large bilateral cavernous carotid aneurysms (CCAs), with a larger multilobulated aneurysmal sac on the right; **(B)** Brain computed tomography (CT) angiography showing large bilateral CAAs measuring approximately 21 × 17 × 16 mm on the right and 18 × 15 × 16 mm on the left; **(C,D)** MR and CT angiography confirming the presence of large bilateral CAAs, unchanged in size from previous scans.

When she presented to our medical attention she was admitted in our department and a brain MRI was undertaken. Axial T1 and T2 weighted images and MRA demonstrated large bilateral CCAs, with a larger multilobulated aneurysmal sac on the right, along with fetal origin of posterior cerebral arteries and small basilar artery (Figure 1A). A CT angiography of brain vessels confirmed the presence of bilateral large CCAs, measuring approximately 21 × 17 × 16 mm on the right and 18 × 15 × 16 mm on the left (Figure 1B).

Surgical resection and endovascular occlusion were held at this time due to high procedural risk in an elderly frail patient.

Based on the hypothesis that cranial nerve involvement in this patient was probably due, at least in part, to thrombosis-related edema and inflammation, an empirical steroid therapy with oral prednisone 1 mg/kg/day was started, followed by slow tapering over the course of 2 months.

MR and CT angiographies performed 4 months later showed no change in the bilateral CCAs compared to previous scans (Figures 1C,D). A follow-up examination showed a severe evolution of the neurological involvement: the patient had complete sixth nerve palsy in the right eye causing convergent strabismus with compensatory head rotation toward the right side, severe ptosis and complete ophthalmoplegia in the left eye. In addition, her left pupil was mydriatic, without light response. Although the size of the aneurysms was unchanged, the patient also started experiencing left periorbital headache.

Because of this progressive deterioration of the clinical conditions, intervention could no longer be delayed. Therefore, the patient underwent endovascular embolization of the left carotid aneurysm with flow diversion. She was treated with dual antiplatelet therapy for 1 month, followed by single antiplatelet drug for other 3 months. At discharge, ptosis in the left eye improved, but complete palsy of oculomotor muscles was still present.

## Discussion

We describe a peculiar case of multiple cranial nerves palsies resulted from compression exerted by bilateral carotid aneurysms in cavernous sinus resulting in fluctuating diplopia that mimicked myasthenia.

Owing to their position in a venous pouch, CCAs grow to a remarkable size before they cause any mass effect related symptoms. These include diplopia, due to involvement of the III, IV and VI cranial nerves independently or in combination, miosis, resulting from involvement of ocular sympathetic innervation, reduction in visual acuity, caused by compressive optic neuropathy, corneal hyperesthesia and trigeminal dysesthesias due to injury of the first / second branch of the trigeminal nerve (2). In addition, pain, ranging from unilateral headache to retro-orbital and facial pain, has been commonly reported.

Involvement of the left third cranial nerve in this patient was atypical because it was initially painless and pupil-sparing; in addition, the fluctuation of symptoms led us to consider myasthenia gravis as a possible diagnosis. With the enlargement of CCAs, a few months later, the patient developed painful complete left ophthalmoplegia, including a complete third cranial nerve palsy with a fixed and dilated pupil, and right sixth nerve palsy, with pain due to mass effect. Compression of third cranial nerve from intracranial aneurysms is not infrequent, but, given the more superficial location of pupillomotor fibers within the nerve fascicle, patients typically manifest with pupillary dilation once ocular motility becomes affected due to third cranial nerve involvement. Within the cavernous sinus, pupillary fibers run along the medial side of the third cranial nerve, therefore they may be spared at presentation of CAAs, being later involved during the aneurysmal growing process (3). This likely explains the pupil-sparing presentation of our patient despite compression on the third cranial nerve. Finally, incomplete third nerve palsy and pupillary sparing may depend on the involvement of the superior division only of the third cranial nerve, which does not include the pupillomotor fibers (4).

A few published cases have reported pupil-sparing third nerve palsy from aneurysms (5–7). Moreover, a recent population-based study revealed that, even if pupil involvement was more likely associated with compressive third cranial nerve palsy, only 64% of patients with compressive third cranial nerve palsy had a pupil involvement, and, only 33% of aneurysms presented pupil abnormalities (8). Specifically, Fang and colleagues found that all three posterior communicating (PCOM) artery aneurysms presented with pupillary involvement, while all five patients with CCAs had a pupil-sparing presentation, with development of pupil involvement over time, similarly to our patient. Pupillary fibers are particularly vulnerable to PCOM aneurysm compression due to their dorso-medial position in the nervous bundle and the anatomical relationship between the subarachnoid portion of the third cranial nerve and the carotid-PCOM artery junction (9). Therefore, PCOM artery aneurysms usually manifest with mydriasis with ocular motility defects, while CCAs can sometimes present with an incomplete third nerve palsy with later progression to pupil involvement and worsening ophthalmoplegia.

It is important to note that complete external ophthalmoplegia with pupil sparing from aneurysmal compression is exceedingly rare and we are aware of only a single case reported in the literature of this (10).

In the same population-study by Fang et al., eye pain and/or headache were common at time of presentation in acquired third cranial nerve palsy, being reported in 60% of all compressive etiologies, and in 78% of aneurysm-dependent third cranial nerve palsies (8). The diagnostic delay in our patient was partially due to the late onset of the periorbital headache; nevertheless, pain is not an accurate symptom to differentiate compressive from microvascular etiologies, since similar prevalence have been reported (11). These findings suggest that ophthalmoparesis without pupil involvement should not exclude nerve compression by an extrinsic mass and thus prompt further investigations, even if there is no pain.

The intermittent nature of diplopia in our patient at first suggested a neuromuscular problem (i.e., ocular myasthenia), rather than a compressive lesion of the third cranial nerve. We can speculate that fatigue in this patient resulted from compromised transmission of axonal action potentials of the compressed cranial nerves, in addition to sustained use of the extraocular muscles. It has been proposed that mechanical nerve irritation may be responsible for the fluctuating symptomatology observed in patients affected by third cranial nerve compression: the alternating opthalmoparesis and third cranial nerve hyperactivity could be ascribed to an inappropriate interchange between neural block and discharge. This has been described in a clinical report by Dayan et al. where the treatment of a third cranial nerve compressing aneurysm caused immediate resolution of third cranial nerve hyperfunction, followed by a rapid but gradual rescue of the third cranial nerve palsy (12).

This type of presentation is consistent with a case of pseudomyasthenia, a term used to define weakness and fatigability of the lids and extraocular muscles without pupils' involvement, not caused by a neuromuscular transmission disorder (13). Although fluctuating ptosis and diplopia are paradigmatic symptoms of ocular myasthenia gravis, they can sometimes be observed in cranial nerve compression, especially if involving third cranial nerve with pupil-sparing. For this reason, CCAs, which can grow and cause oculomotor nerve compression with pupil sparing, may be mistaken for a neuromuscular junction disorder.

Pseudomyasthenia was also found to be a clinical manifestation of posterior communicating artery, basilar artery aneurysms, and carotid aneurysms (14–16). Mindel et al. reported the case of two patients with ophthalmoparesis and intermittent diplopia from carotid aneurysms mimicking ocular myasthenia (16). Both patients also presented with increased prolactin level and one of them was treated with piridostigmine for a few months, without clinical benefit. Assessment for myasthenia, including EMG with repetitive stimulation, edrophonium test, and specific antibody titers, were negative, while brain CT and brain angiography demonstrated the presence of large intracranial carotid aneurysms. A case of fluctuating ptosis and pupil-sparing diplopia, with later development of anisocoria, was described in a 67-year-old patient with a saccular aneurysm of cavernous and supraclinoid segment of the left internal carotid artery (17).

In addition to ocular myasthenia, because of their position in the skull and proximity with other structures, like pituitary gland, bilateral CCAs can simulate other diseases or conditions. For instance, they can imitate pituitary adenomas when they extend into the sella. Torres at al. described the case of an 82-years-old woman with a history of hypothyroidism, complaining headache, nausea, vomiting and diplopia (18). The patient also had arterial hypotension and hyponatremia (consistent with Addison disease), as well as hyperprolactinemia, presenting with classic signs and symptoms of pituitary apoplexy. Brain imaging showed an intrasellar mass simulating a large pituitary adenoma, and she was treated with high-dose hydrocortisone. Thanks to histological analysis and subsequently brain angiography, the intracranial mass was found to be bilateral aneurysms in the cavernous portion of the internal carotid artery. Previous cases of bilateral CCAs manifesting with pituitary apoplexy were described, and also the association with pituitary tumors and carotid intracranial aneurysms exists (19, 20).

In order to assess the frequency, clinical presentation and management of bilateral carotid artery aneurysms of the cavernous tract, we conducted an extensive review of the literature and we found several cases of bilateral idiopathic unruptured CCAs (Table 1).

**Table 1:** Bilateral idiopathic unruptured cavernous carotid aneurysms: review of the literature.

**Reference**	**Age (years) and gender**	**Presentation**	**Neurological examination**	**Neuro radiological findings**	**Additional findings**	**Treatment**	**Outcome**
21, Hurteau, personal communication	47, female	Sudden onset of left supra- and periorbital headache and medial deviation of the left eye	Left VI opthalmoplegia and ptosis, left supraorbital and corneal hypoesthesia, left dilated pupil; blurred vision in the left eye	Bilateral CCAs		Ligation of left carotid artery proximal and distal to the aneurysm	Left eye movement restored but impairment of fine right-hand movements
(22)	54, female	Sudden onset of inward deviation of the right eye followed by intermittent right retro-orbital headache	Right palsy of external, superior and inferior recti muscles, right dilated fixed pupil, absence of right corneal reflex, reduction of visual acuity in the right eye; right V_3_ tactile hypoesthesia, right paresis of muscles of mastication	Right large multilobulated saccular cavernous aneurysm and a smaller left cavernous aneurysm	Poor filling of the anterior cerebral artery	Surgery was ruled out because of higher risk of seizures recurrence; patients was treated with anticonvulsant medication	9 months later additional neurological findings were paresis of elevation of the left eye and increased loss of vision in the right eye
(23)	25, female	Abrupt left VI cranial nerve palsy, followed by left frontal headache and III and IV cranial nerves palsy	Left III, IV and VI cranial nerves palsy with reduction of the left corneal reflex, left altitudinal hemianopsia, reduction of visual acuity in the left eye	Bilateral large CCAs, with partial thrombosis in the left aneurysmal sac	Occlusion of left superior ophthalmic vein		
(24)	42, female	Diplopia	Ptosis and ophtalmoparesis	Bilateral CAAs		Left carotid ligation and right partial carotid occlusion 3 years later	Recovery except for a slight right ptosis and a limitation of vertical gaze
(25)	41, female	Diplopia	Complete left ophthalmoplegia and impending right ophthalmoplegia	Bilateral giant CCAs		Right carotid ligation followed 1 week later by subtotal left carotid occlusion	Dramatic recovery
(16)	74, female	6-month history of intermittent diplopia in all gaze directions	Right hypertropia on sustained upgaze or right, and left hypertropia on sustained down gaze or left	Bilateral CAAs	Increased serum prolactine		
(16)	65, female	Intermittent diplopia that became more frequent and occasional headache above the left eye	Bilateral ptosis more marked on the left, the left eye only had lateral movements	Bilateral CAAs, larger on the right	Slight elevation of serum thyroxine level	Right EC-IC bypass followed by Silverstone clamp occlusion of the right internal carotid artery	No change in patient neurological status
(26)	16, male	4-month history of diplopia on upward gaze	Left lateral rectus palsy, limitation of elevation of the right eye, with a partial ptosis and an efferent pupillary defect	Bilateral giant CCAs	Small anterior communicating artery aneurysm	No surgical intervention; management of arterial hypertension	No signs of neurological progression within 28 months
(27)	4, male	Strabismus	Bilateral III cranial nerve palsies	Bilateral CCAs	Bilateral large posterior communicating arteries, patent anterior communicating artery	Endovascular treatment with mechanically detectable coils	III cranial nerve palsies resolved after 3 months
(28)	54, male	Double vision, left facial dysesthesia and ophthalmic pain	Left severe III cranial nerve palsy	Giant left CCA with subarachnoid extension, large right CCA	Bilateral large VAAs	Selverstone clamp on the left carotid artery, followed by permanent ligation	Left III cranial nerve palsy resolved within several months; 2 years later SAH from VAA rupture leading to patient's death
(29)	76, male	Vertical diplopia, followed by left retroocular headache	Left mild pupillary dilation, reduction in left eye downward movements, mild left palpebral ptosis	Giant bilateral CCAs	Anomalous origin of the left internal carotid artery, bilateral renal artery stenosis	No treatment given spontaneous subsidence of symptoms, patient's age and clinical condition	Spontaneous subsidence of symptoms
(30)	85, female	Acute worsening diplopia	Left IV and VI cranial nerves palsy	Bilateral CCAs		Occlusion of the left eye; no surgical treatment given patient's age	
(31)	52, female	Right-sided ptosis and facial pain on V_1_ territory	Complete right ophthalmoplegia, right-sided non-reflectic mydriasis, corneal hypoesthesia	Mirror giant CCAs	Multiple intra- and extracranial aneurysms and a third cervical carotid artery aneurysm	Patient refused surgery	Foix syndrome improved
(32)	73, female	Worsening diplopia	Bilateral paresis of lateral rectus muscles	Bilateral CCAs		No treatment other than orthopyic correction of the diplopia and management of arterial hypertension	
(33)	26, female	Transient worsening diplopia and headache	Left III cranial nerve palsy	Bilateral giant CCAs		Coil embolization using a detachable microcoil at both sides with a distance of 3 months	Complete recovery without diplopia
(34)	28, female	Double vision and left blepharoptosis	Left III cranial nerve palsy and V_3_ hypoesthesia; then right V_3_ hypoesthesia and ptosis	Bilateral CCAs		EC-IC bypass with PAO at both sides with a distance of 10 months	Left III cranial nerve palsy resolved within 1 month from the first surgery; gradual improvement of cranial nerves palsy within 2 months from the second surgery
(35)	65, female	Double vision	Left III and VI cranial nerves palsy	Left giant and right large CCAs		Left ICA proximal ligation with flow bypass; stent-assisted endovascular coil embolization at the right side after 3 months	Favorable outcome

*CCA(s), Cavernous carotid aneurysm(s); VAA(s), Vertebral artery aneurysm(s); ICA, internal carotid artery; SAH, subarachnoid hemorrhage; EC-IC, extracranial-intracranial; PAO, patent artery occlusion*.

In conclusion, bilateral CCAs represent a rare medical condition that can mimic other disorders, such as myasthenia gravis or pituitary adenoma. The presence of fluctuating diplopia and the lack of pupil involvement are not sufficient to exclude compressive etiology, which should always be suspected, especially in older patients. Lesions compressing the oculomotor nerve can sometimes present with pupil-sparing partial ophthalmoplegia. In addition, the presence of pain does not accurately discriminate between compressive and ischemic lesions.

Although infrequent, cavernous aneurysms are a potentially treatable cause of extraocular muscle palsy. Therefore, we advise investigation including brain MRI and MRA with detail given to the intracranial vessels for patients with multiple cranial nerve palsies involving the extraocular muscles, even if there is pupil sparing.

## Ethics statement

The case report has been performed in accordance with the ethical standards laid down in the 1964 Declaration of Helsinki and its later amendments. Informed written consent was obtained from the participant prior to the inclusion.

## Author contributions

All the authors took care of patient management and made decisions about patient treatment. DG and IF conceived the idea, revised all the literature and wrote the manuscript. LV collected the clinical data. GP collected the clinical data and performed the endovascular procedure. CC analyzed and interpreted brain imaging. LT analyzed ophthalmological data and made revisions to the Discussion section by adding important intellectual content. RB, EM, FM, and AG collected the clinical data and contributed to the writing of the manuscript. NB, GC, and SC contributed to the revision of the manuscript, read and approved the submitted version.

### Conflict of interest statement

The authors declare that the research was conducted in the absence of any commercial or financial relationships that could be construed as a potential conflict of interest. The reviewer MA and handling Editor declared their shared affiliation at the time of the review.
